# Anterior chamber enhancement predicts optic nerve infiltration in retinoblastoma

**DOI:** 10.1007/s00330-022-08778-4

**Published:** 2022-05-07

**Authors:** Katerina Deike-Hofmann, Paula von Lampe, Maija Eerikaeinen, Saskia Ting, Sabrina Schlüter, Heinz-Peter Schlemmer, Nikolaos E. Bechrakis, Michael Forsting, Alexander Radbruch

**Affiliations:** 1 Department of Radiology, German Cancer Research Center, DKFZ, Heidelberg, Germany; 2 Department of Neuroradiology, University Hospital Bonn, Bonn, Germany; 3 Clinical Neuroimaging Group, German Center for Neurodegenerative Diseases, DZNE, Bonn, Germany; 4 Department of Radiology, University Hospital Essen, Essen, Germany; 5 Department of Pathology, University Hospital Essen, Essen, Germany; 6 Department of Ophthalmology, University Hospital Essen, Essen, Germany

**Keywords:** Gadolinium, Retinoblastoma, Glymphatic system, Optic nerve, Magnetic resonance imaging

## Abstract

**Objectives:**

As described recently, intravenously injected gadolinium-based contrast agent (GBCA) penetrates into the anterior eye chamber (AC) and is drained from the retina to the distal optic nerve (ON) along perivascular spaces, which serves retinal homeostasis and was termed the orbital glymphatic system (GS). Independently, AC enhancement predicted ON infiltration, a major risk factor for advanced retinoblastoma (RB), in a small RB patient cohort. We aimed to review the supposed imaging biomarker for ON infiltration in a large RB cohort and with respect to the recently described orbital GS.

**Methods:**

This IRB–approved retrospective single-center study encompassed 539 orbital MRIs performed with an orbital coil and with the children under general anesthesia. Differences of signal intensity ratios (∆SIRs) of the AC to the lens were determined between non-contrast and GBCA–enhanced T1-weighted images and were correlated with histopathologic presence of ON infiltration.

**Results:**

∆SIR of the RB eye was an independent, significant predictor for ON invasion in multivariate analysis with adjustment for tumor size (*p* < 0.05) and increased with infiltration level.

**Conclusions:**

GBCA enhancement of the AC predicts ON infiltration. This might be caused by impairment of the orbital glymphatic system, which is supposed to clear toxic metabolites from the retina to the postlaminar ON. In RB with ON infiltration, this efflux path is likely to be inhibited, which is supposed to result in disturbed retinal homeostasis, release of vascular endothelial growth factor, and iris neovascularization, which increases penetration of GBCA into the AC.

**Key Points:**

• *Infiltration of the optic nerve can be predicted by anterior chamber enhancement after intravenous MRI contrast agent administration.*

• *Increased anterior chamber enhancement in retinoblastoma with optic nerve infiltration might result from dysfunction of the orbital glymphatic system with disturbance of retinal homeostasis and consecutive iris neovascularization.*

**Supplementary Information:**

The online version contains supplementary material available at 10.1007/s00330-022-08778-4.

## Introduction

In children suffering from retinoblastoma (RB), optic nerve (ON) infiltration is a major risk factor for advanced disease [1, 2]. As RB are “no-touch lesions,” due to the high risk of tumor seeding, therapy is initiated without previous histological examination and pretreatment staging MRI plays a key role in the diagnostic workup of RB. Orbital MRI needs to identify risk factors of advanced disease such as ON infiltration as they guide therapy decisions. Specifically, while bulb enucleation remains the principal therapy, vision-preserving therapy is highly desirable in the very early disease stage [3]. Furthermore, knowledge on ON infiltration determines the security margin with which the distal ON is resected when enucleation has to be performed [4]. Hence, it is essential for pretreatment MRI to identify ON infiltration, but the diagnostic accuracy is highly variable, with reported sensitivities ranging from 50 to 73% [5–11].

Recently, a correlation between ON infiltration and gadolinium-based contrast agent (GBCA) enhancement of the anterior eye chamber was reported in a small cohort of RB patients [12].

The orbital glymphatic system functions in accordance to the glymphatic system of the brain and mediates perivascular clearance of toxic metabolites from the retina to the distal ON to maintain retinal homeostasis [13]. Disturbance of retinal homeostasis is known to result in neovascular glaucoma and iris neovascularization [14–16], which might explain the observed increase of GBCA penetration into the AC in RB with ON infiltration reported by Galluzzi et al. Consistently, it was reported that AC enhancement was accompanied by the presence of neovascular processes of the anterior eye segment [5, 12, 17].

While it is known that the orbital glymphatic pathway can be visualized with GBCA–enhanced heavily T2-weighted (hT2) MRI, which is highly sensitive to GBCAs in fluids [18–21], standard T1-weighting usually does not reveal the small amount of GBCA crossing the blood-ocular barrier after intravenous injection. However, in this study, we consulted a large RB cohort that was investigated with bilateral orbital coils under anesthesia. In this specific setting, which allows for high-resolution T1-weighting with suppression of eye motion artifacts and provides T1-weighting with identical sequence parameters prior to and following GBCA injection, T1-weighting was previously shown to reveal the slight signal increase caused by intravenously injected GBCA penetrating into the AC of healthy eyes [22].

In this study, we aimed to review the suggested imaging biomarker of ON infiltration in a large cohort of a national RB center and put results into context of the recently described orbital glymphatic system.

## Patients and methods

### Patients

**Fig. 1:**
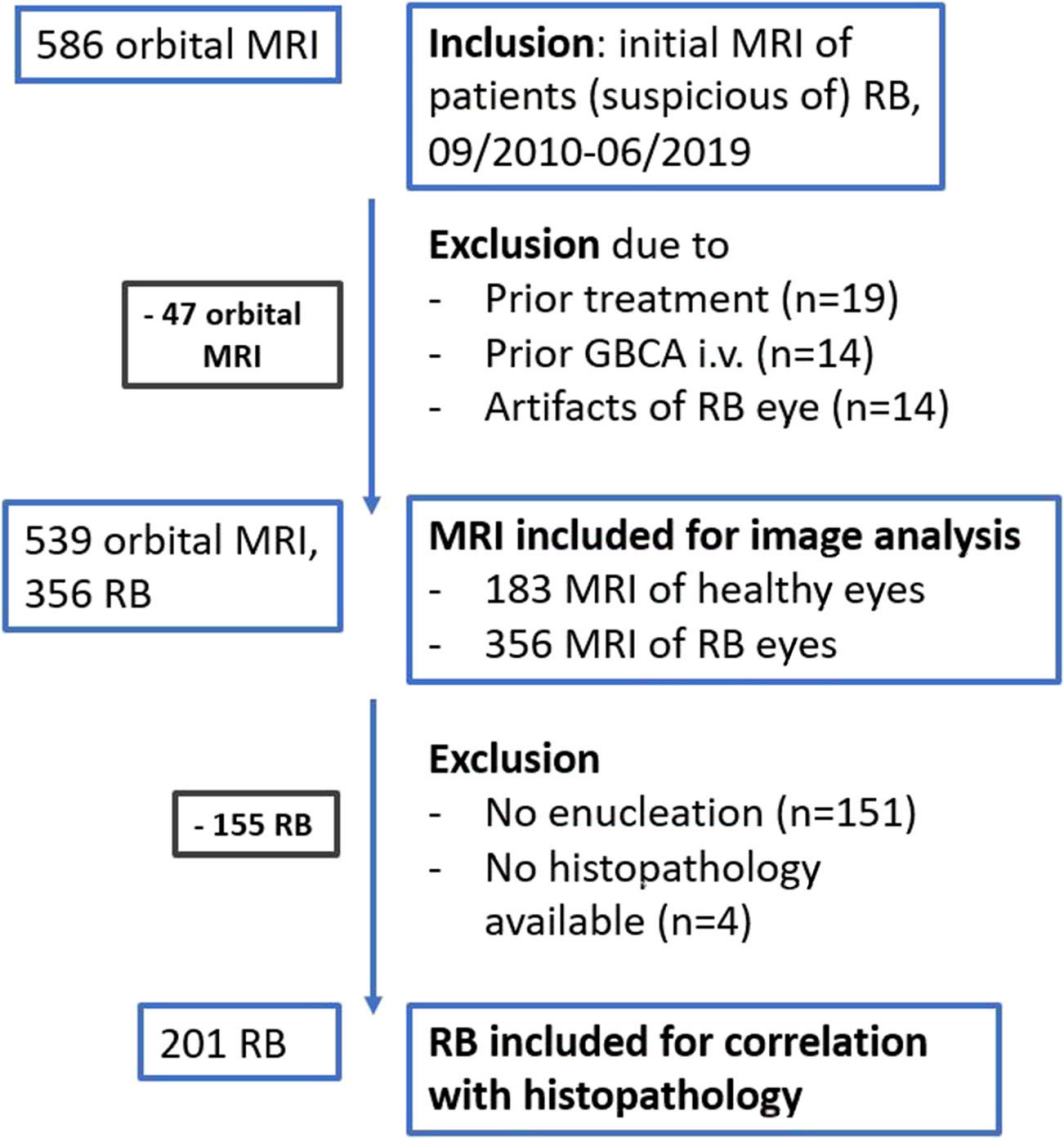
Flow chart with inclusion and exclusion criteria. Abbreviations: RB, retinoblastoma; GBCA, gadolinium-based contrast agent

**Fig. 2:**
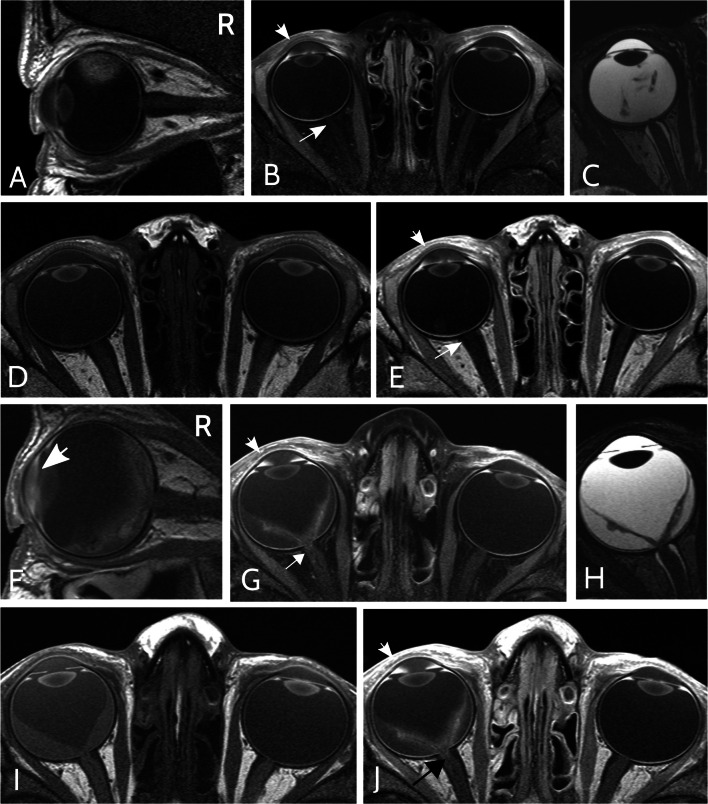
Example of orbital MRI of retinoblastoma with optic nerve infiltration. Sagittal (**A**, **F**) and axial fat-saturated (**B**, **G**) T1-weighted MRI post injection of a gadolinium-based contrast agent (GBCA). Axial T2-weighed (**C**, **H**) and axial native (**D**, **I**) as well as GBCA–enhanced (**E**, **J**) T1-weighted MRI. (**A**–**E**) Orbital MRI sequences of a right-sided retinoblastoma (RB) with prelaminar optic nerve (ON) infiltration in a 36-month-old boy. While the right anterior eye chamber (AC) shows GBCA enhancement (short arrow in **B** + **E**), the optic nerve does not show GBCA enhancement (long arrow in **B** + **E**). Subsequent enucleation and histological analysis revealed prelaminar ON infiltration. (**F**–**J**) Orbital MRI of a right-sided RB with postlaminar ON infiltration in a 40-month-old boy. Both the right AC and distal ON show pronounced GBCA enhancement (**G**, **J**, short and long arrow, respectively).

Written informed consent was waived due to the retrospective character of this IRB–approved study. All initial orbital MRI of patients suspicious of RB which were performed at our institution between September 2010 and June 2019 were reviewed (*n* = 586). MRI were excluded if therapy had already started, any GBCA injection had been performed within 8 weeks prior to the respective orbital MRI, or insufficient image quality impeded image analysis, e.g., due to motion artifacts. Finally, 539 orbital MRI were included, 356 of RB eyes and 183 of healthy eyes. The flow chart in Fig. 1 presents the number of included orbital MRI with inclusion and exclusion criteria. Two examples of orbital MRI are provided in Fig. 2.

### MRI protocol

All patients were assessed at the same 1.5-T MRI scanner (MAGNETOM Aera, Siemens Healthineers) using a 4-cm loop coil. Identical T1-weighted images (repetition time 583 ms, echo time 16 ms, flip angle 90°, voxel size 0.2 × 0.2 × 2.0 mm, field of view (FoV) read 80 mm, FoV phase 131.3%, 20 slices, distance factor 20%, acquisition time 7:53 min) were obtained prior to and 12:21 ± 2:36 minutes (mean ± standard deviation (SD)) post injection of a standard dose of gadoterate dimeglumine (0.1 ml/kg body weight). MRI was performed with the children in a state of general anesthesia induced by propofol (1 ml/kg body weight, 1%).

For 47 RB eyes in 38 children, signal intensity ratios (SIRs) were calculated from T1-weighted images with and without active prescan normalization to assess the influence of prescan normalization on SIRs.

### Image analysis

Every case was assessed by either of two readers with 1 and 3 years of experience in neuroradiology, who conducted image analysis on an accredited workstation after a pre-reading session with teaching by a neuroradiologist with 10 years of experience.

Signal intensities (SIs) of the AC and lens were extracted via region-of-interest (ROI) measurements with ROIs drawn in the AC and lens on the most representative axial slice (supplementary fig. e-1).

The difference in AC-to-lens SIR (∆SIR) between pre- and post-GBCA–enhanced scans was determined as follows:

$$ \mathrm{AC}\hbox{-} \mathrm{to}\hbox{-} \mathrm{lens}\ \Delta \mathrm{SIR}=\left(\mathrm{SI}\left({\mathrm{AC}}_{\mathrm{post}}\right)/\mathrm{SI}\left({\mathrm{lens}}_{\mathrm{post}}\right)\right)-\left(\mathrm{SI}\left({\mathrm{AC}}_{\mathrm{pre}}\right)/\mathrm{SI}\left({\mathrm{lens}}_{\mathrm{pre}}\right)\right). $$

Tumor size was classified with a 6-point ordinal scale on the basis of the axial plane with the most prominent tumor presence. Affection of the entire globe was scored as a 6 (supplementary fig. e-2). If tumor expansion on the sagittal plane suggested a substantially different score, the score was adapted. Histopathology of enucleated RB eyes was assessed by chart review.

### Statistical analysis

Statistical analysis was performed with the software package R (R Foundation for Statistical Computing, version 3.5.2, 2018-12-20). Significance was set at the *p* < 0.05 level. Mann-Whitney *U* test was performed to compare ∆SIRs of healthy and RB eyes, as well as ∆SIRs of different ON infiltration levels. Wilcoxon test was conducted to compare pre- and post-contrast SIRs.

Multivariate logistic regression with adjustment for tumor size was performed to investigate if ∆SIR can predict histopathological tumor features. Logistic regression was chosen, because we aimed to not only correlate ∆SIR and histopathology, but also to investigate the value of ∆SIR in predicting a dichotomous target variable, i.e., presence or absence of histopathological infiltration. As only ∆SIR but not the target variable, i.e., ON infiltration status, depends on patient age and post-injection (p.i.) time, age and p.i. time were not taken into account as confounders.

## Results

### Patient characteristics

Patient characteristics are summarized in Table 1. Five hundred thirty-nine orbital MRI of 270 consecutive treatment-naïve patients with uni- or bilateral RB were included in the study. Analysis encompassed 356 eyes harboring RB and 183 healthy eyes in 183 children with unilateral (67.8%) and 87 patients with bilateral RB (32.2%). In one case of bilateral RB, only one RB eye could be assessed as the other one had already been enucleated. The healthy eyes of patients with unilateral RB were part of a previously published study about GBCA dynamics in healthy infantile eyes. Average age (range) of the patients was 18.9 (0–91) months. One hundred seventeen girls (43.3%) and 153 boys (56.7%) were included. Mean (range) serum creatinine level was 0.43 (0.19–0.77) mg/dl. The International Classification of Retinoblastoma (ICRB) score was extracted from the tumor board documentation, if available (available for 322 of 356 RB eyes; results are presented in Table 1). Two hundred five of 356 RB eyes were enucleated (57.6%); for 201 of 205 enucleated eyes, a histopathology report could be extracted from the clinical documentation system (compare Fig. 1).

**Table 1:** Patient characteristics

Characteristic	Number
Patients	
Total	270
With unilateral RB	183
With bilateral RB	87
Orbital MRI	
Total	539
With RB	356^1^
From unilateral RB	183
From bilateral RB	173^1^
Without RB (i.e., from unilateral RB)	183
Age [months], mean (range)	18.9 (0.0–91.0)
Gender [female %]	43.3%
Serum creatinine [mg/dl] mean (range)	0.43 (0.19–0.77)
ICRB score^2^	
Group A	16
Group B	71
Group C	13
Group D	47
Group E	175
NA	34
ON infiltration	
No	94 (46.8%)
Prelaminar	73 (36.3%)
Laminar	20 (10.0%)
Postlaminar	14 (7.0%)
Central vessel infiltration	
Yes	2 (1.0%)
No	198
NA	1

*ICRB* International Classification of Retinoblastoma, *RB* retinoblastoma, *ON* optic nerve

^1^Of one patient with bilateral RB, only one RB was included, because the other eye had already been enucleated

^2^ICRB score was taken from the tumor board documentation

### Comparison of AC-to-lens SIRs

SIRs derived from scans with and without active prescan normalization showed a strong linear correlation (*r*_Pearson_ > 0.99, *p* < 0.001, supplementary fig. e-3). Therefore, assessment of orbital MRI with active prescan normalization was deemed to be valid.

Median SIR of contrast-enhanced scans was significantly greater than that of native scans in RB eyes (median SIR_pre_ (IQR) = 76.8 (63.5–92.1), median SIR_post_ (IQR) = 105.8 (82.5–170.4), Wilcoxon test, *p* < 0.001). SIRs and ∆SIRs are presented in Table 2.

**Table 2:** Summary of signal intensity ratios (SIRs) and differences in signal intensity ratios (∆SIRs)

	Median	Interquartile range (IQR)	*p* value
SIR_pre_	76.8	63.5–92.1	< 0.001
SIR_post_	105.8	82.5–170.4	
∆SIR_RB_	0.23	0.10–0.60	< 0.001
∆SIR_healthy_	0.08	0.05–0.13	
∆SIR_RB_, ON infiltration			
Yes (*n* = 107)	0.62	0.18–1.21	0.001
No (*n* = 94)	0.27	0.12–0.52	
∆SIR_RB_, ON infiltration			
None (*n* = 94)	0.27	0.12–0.52	–
Prelaminar (*n* = 73)	0.50	0.14–1.13	0.03
Laminar (*n* = 20)	0.62	0.27–1.32	0.004
Postlaminar (*n* = 14)	0.85	0.50–1.22	0.01
∆SIR_RB_, ON infiltration			
None + prelaminar	0.35	0.12–0.91	–
Postlaminar	0.85	0.50–1.22	0.06

*∆SIR* differences of signal intensity ratios of the anterior chamber to the lens, *SIR* signal intensity ratios of the anterior chamber to the lens, *SIR*_*pre*_ SIR prior to contrast agent injection, *SIR*_*post*_ SIR post contrast agent injection, *RB* retinoblastoma, *ON* optic nerve

### Comparison of AC-to-lens signal intensity ratio differences

Median ∆SIR of RB eyes was significantly greater as compared to that of healthy eyes (median ∆SIR_RB_ (IQR) = 0.23 (0.10–0.60), median ∆SIR_healthy_ (IQR) = 0.08 (0.05–0.13), Mann-Whitney *U* test, *p* < 0.001).

**Fig. 3:**
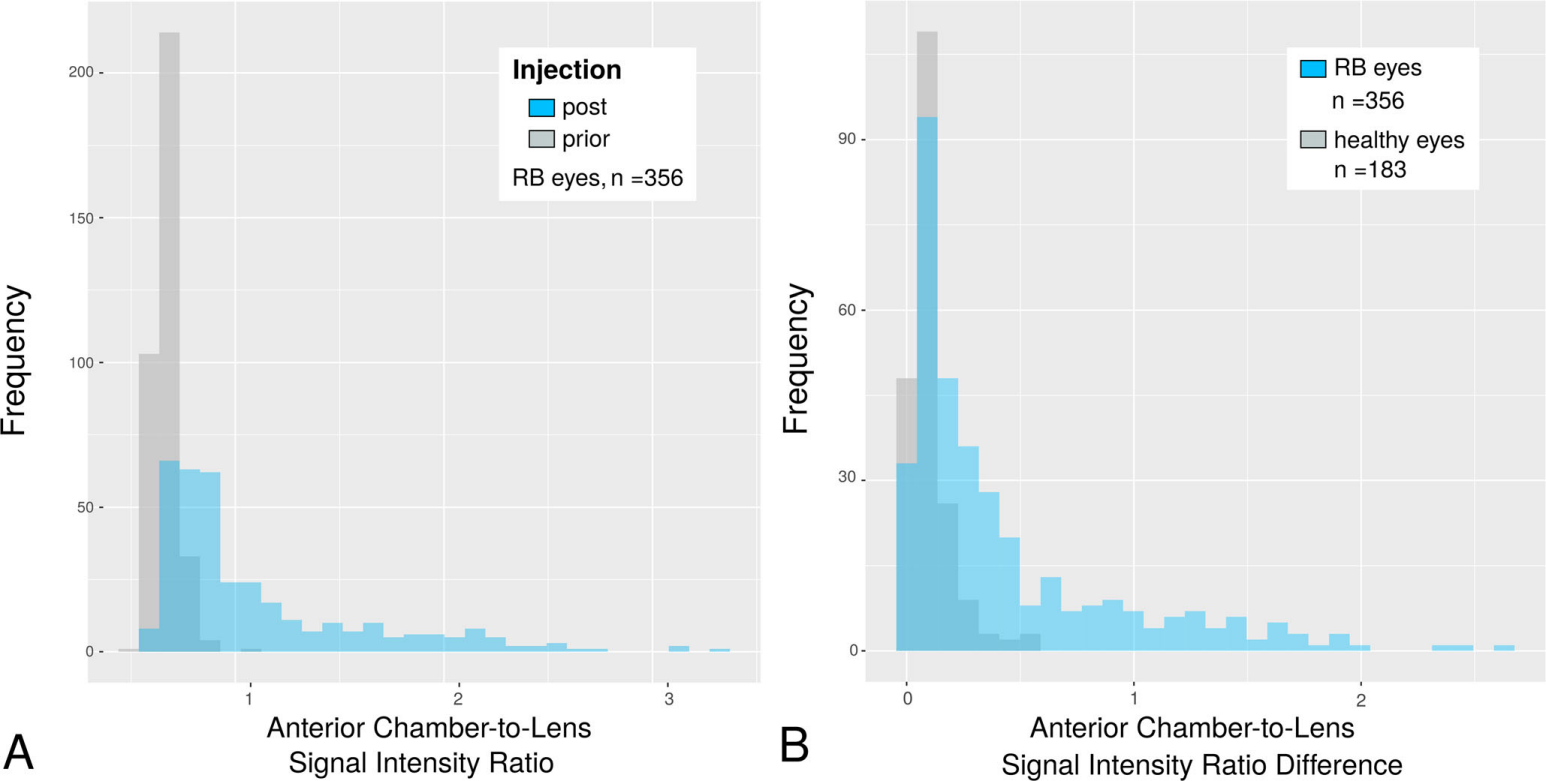
**A** Comparison of histograms of anterior chamber (AC)-to-lens signal intensity ratios (SIRs) prior to and following gadolinium-based contrast agent (GBCA) injection in eyes harboring retinoblastoma (RB, *n* = 356). Mean post-injection time (± standard deviation) was 12:21 ± 2:36 min. Mean SIR post injection was significantly higher than prior to injection indicating GBCA penetration into the AC (*p* < 0.001). **B** Comparison of difference of AC-to-lens SIRs (∆SIRs) between healthy eyes (*n* = 183) and eyes affected by RB (*n* = 356). Mean ∆SIR of RB eyes was significantly higher compared to that of healthy eyes indicating increased GBCA accumulation in eyes with RB (*p* < 0.001)

Figure 2 presents examples of the different MRI sequences of the orbital MRI protocol that allow for comparison of native and GBCA–enhanced ACs as well as of RB and healthy eyes in a patient with prelaminar and a patient with postlaminar ON infiltration. Histograms comparing SIRs and ∆SIRs are presented in Fig. 3.

Qualitative assessment of the AC revealed a slightly visible SI increase adjacent to the ventral iris with a maximum in the iridocorneal angle in 238 of 356 RB eyes (66.9%) and 38 of 183 (20.8%) healthy eyes (Fig. 2(I + J), and supplementary fig. e-1F).

### Correlation of AC enhancement and ON infiltration

In 201 of 205 enucleated eyes, histopathology with information about ON infiltration was available. One hundred seven RBs depicted ON involvement (53.2%). Median ∆SIR in RB eyes with ON involvement was significantly greater than that of RB eyes without ON infiltration (*p* = 0.001).

**Fig. 4:**
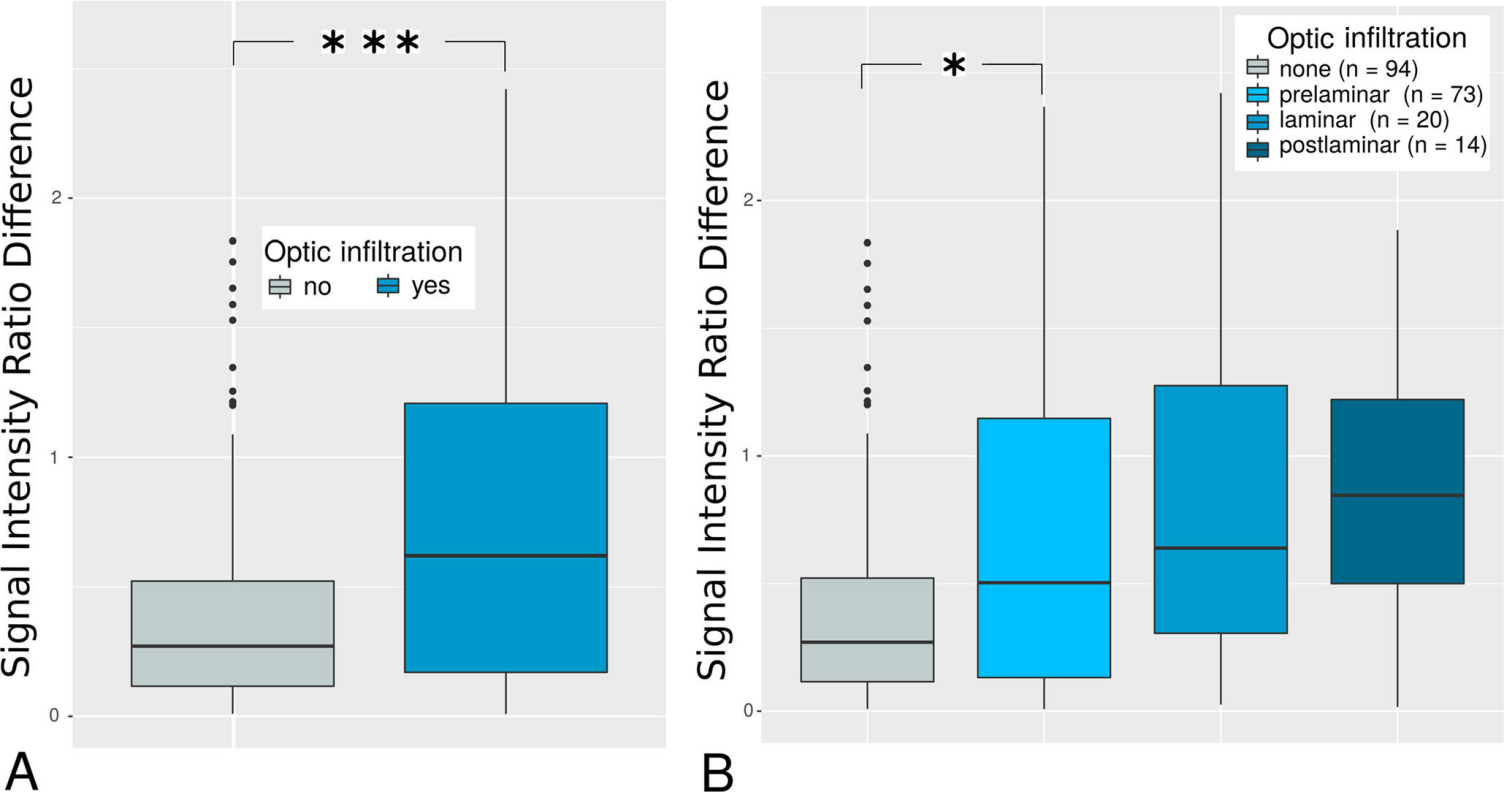
Boxplots of anterior chamber-to-lens signal intensity ratio differences (∆SIRs) depending on the optic infiltration level. The number of asterisks represent the level of significance (*p < 0.05, **p < 0.01, ***p < 0.001). **A** Differences of anterior chamber-to-lens signal intensity ratios (∆SIRs) were significantly higher in retinoblastoma (RB) eyes with infiltration of the optical nerve (*n* = 107) compared to absence of optic nerve infiltration (*n* = 94, *p* = 0.001), indicating gadolinium-based contrast agent (GBCA) enhancement in the anterior chamber (AC) dependent on optic infiltration status. **B** ∆SIRs increased gradually with degree of optic nerve infiltration. Even prelaminar infiltration showed a significantly greater ∆SIR compared to absence of ON infiltration (*p* = 0.03)

Seventy-three RBs showed prelaminar (36.3), 20 laminar (10.0%), and 14 postlaminar (7.0%) infiltration of the ON. Degree of AC enhancement increased with ON infiltration level with a significantly higher median ∆SIR of prelaminar ON infiltration compared to absence of ON infiltration (*p* = 0.03, Fig. 4(B)). Furthermore, median ∆SIR of RB eyes with postlaminar ON involvement was compared to that of all RB eyes with either no or prelaminar ON infiltration (*p* = 0.06).

Multivariate logistic regression with adjustment for tumor size revealed that ∆SIR was an independent positive predictor for ON invasion with doubled infiltration risk per unit change of ∆SIR (OR_∆SIR_ (IQR) = 1.9 (1.1–3.6), *p*_∆SIR_ = 0.03). In turn, with a maximum observed ∆SIR of 2.6, this patient’s risk for optic infiltration was five times as high as the risk of the patient with the lowest observed ∆SIR of zero.

In addition, tumor size was a significant predictor for ON infiltration (OR_tumor size_ (IQR) = 1.3 (1.0–1.6), *p*_tumor size_ = 0.04).

## Discussion

The current study revealed that the degree of GBCA enhancement in the anterior eye chamber is predictive for optic nerve infiltration—one of the major risk factors for advanced disease in children suffering from retinoblastoma.

This might prospectively be exploited as a diagnostic imaging biomarker and serves as an example of how knowledge about the cerebral excretion pathway of GBCAs might yield diagnostic value.

### The orbital glymphatic system

**Fig. 5:**
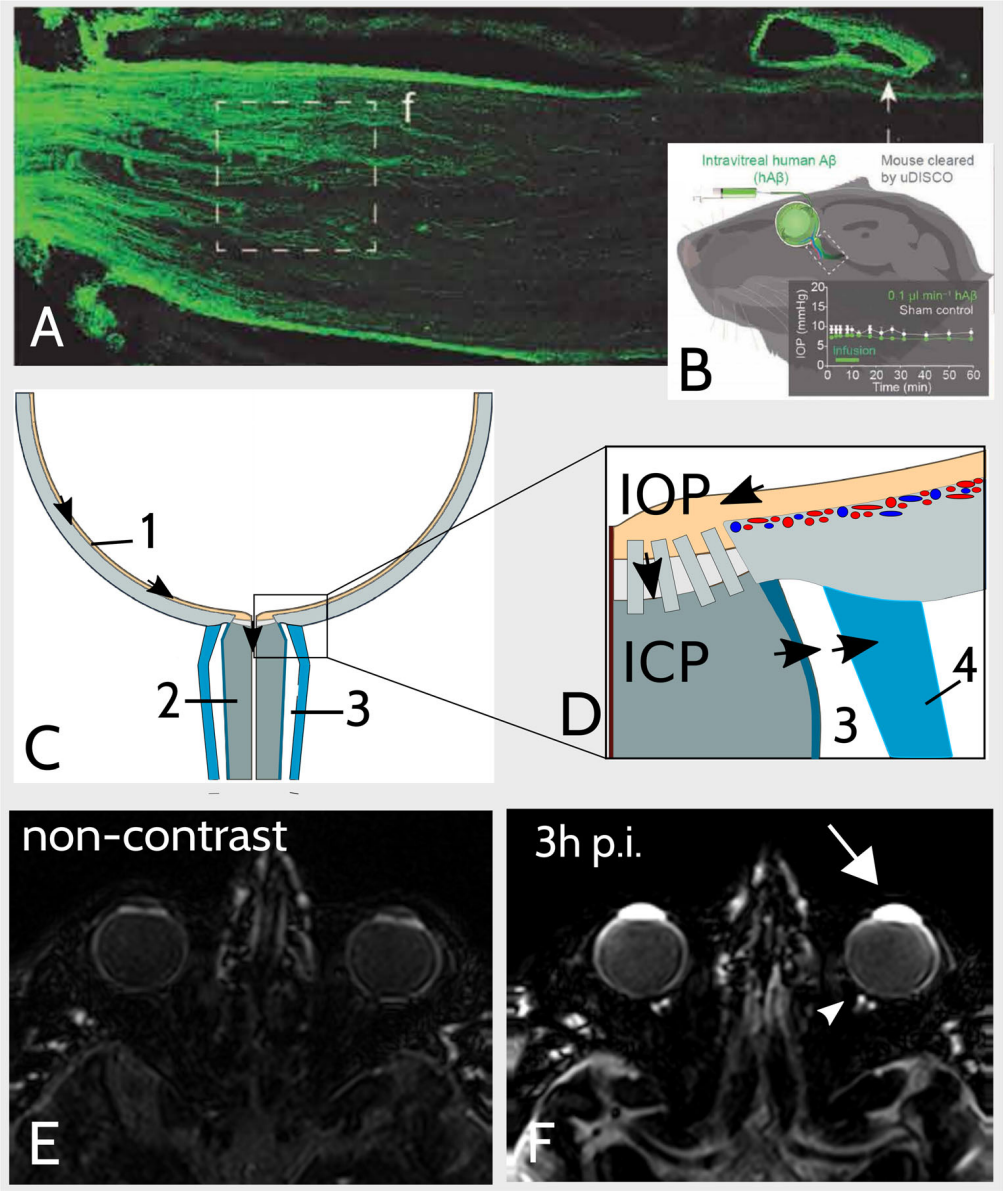
Illustration of the orbital glymphatic theory. **A**, **B** Fluorescence imaging depicted orbital glymphatic flow from the retina to the postlaminar optic nerve (ON). Images were extracted from Wang et al [13] and show that intravitreally injected fluorescent-labeled human β-Amyloid (hAβ-647) is drained from the retina to the postlaminar ON via perivenous spaces (PVS) in rodents (**A**). Remarkably, tracer accumulated in the dural lining of the nerve as well as in cervical lymph nodes post injection (p.i.). Intraocular pressure remained constant during injection (**B**). **C**, **D** Schematic illustration of orbital glymphatic flow. The orbital glymphatic model comprises perivenous fluid and waste drainage (black arrows) from the retina (1) to the postlaminar ON (2) and subarachnoid space (SAS) surrounding the ON (3) driven by the intraocular-intracranial pressure difference (IOP, ICP). Drainage from the SAS is mediated by meningeal lymphatics in the dura mater (4). **E**, **F** Orbital gadolinium-based contrast agent (GBCA) distribution following intravenous injection. The here-shown images were obtained by a heavily T2-weighted fluid-attenuated inversion recovery sequence (hT2w) which is highly sensitive to GBCAs in fluids while suppressing tissue signal (**E**). Three hours post intravenous GBCA injection (**F**) enhancement of ocular structures (inter alia anterior chamber, white arrow) is accompanied by enhancement of the SAS surrounding the postlaminar ON (white arrowhead). This suggests that intravenous GBCA injection enables visualization of the orbital glymphatic flow

While AC hyperintensity has been recognized in patients with RB before, studies on brain clearance imaging revealed that GBCAs penetrate into the AC physiologically and questioned the value of AC hyperintensity as a potential imaging biomarker [5, 12, 19, 22]. Furthermore, AC enhancement of the healthy eye is accompanied by enhancement of the vitreous and of the subarachnoid space (SAS) surrounding the postlaminar ON on heavily T2-weighted FLAIR MRI 3 h post GBCA injection (Fig. 5) [19]. Accordingly, apart from uveoscleral drainage and outflow via the Schlemm’s canal, recently, an additional posterior efflux pathway of intraocular fluid into the postlaminar ON was suggested, which is supposed to be mediated by the trans-laminar cribrosa pressure difference (TLCPD) [13, 23]. This posterior efflux path of ocular fluid was shown to follow the principles of the glymphatic system and therefore termed the orbital glymphatic system. Intravitreal injection of fluorescent tracers was followed by perivascular enhancement of vessels in both the retina as well as ON, and communication between perivascular spaces within the ON and the SAS surrounding the ON was reported [23]. Finally, reabsorption of CSF tracer from the SAS of the ON is supposed to be carried out by meningeal lymphatics located in the dura ensheathing the ON [24–26].

In analogy to the brain glymphatic system, which mediates interstitial-fluid / cerebrospinal-fluid (CSF) exchange and serves waste clearance from the brain, ocular-fluid / CSF exchange serves nutritional function and metabolite clearance from the retina. As the retina is one of the most metabolically active tissues in the human body, orbital glymphatic function might be crucial to maintain retinal homeostasis and function.

**Fig. 6:**
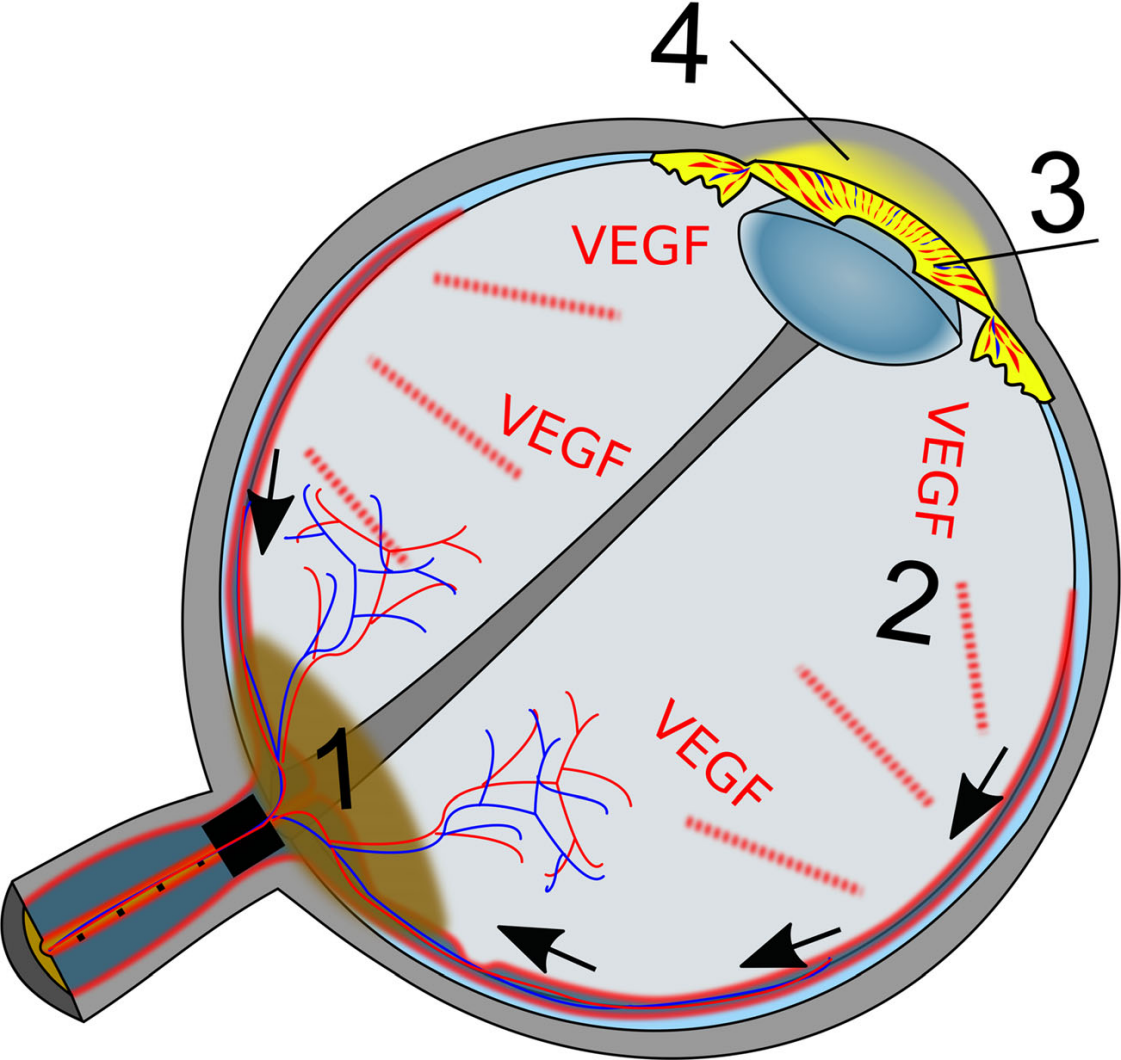
Schematic depiction of hypothesis about correlation between anterior chamber (AC) enhancement and optic nerve (ON) infiltration in eyes affected by retinoblastoma (RB). Infiltration of the ON by RB (1) impedes perivenous glymphatic flow (black arrows) from the retina to the postlaminar ON. As the orbital glymphatic flow is supposed to serve fluid and waste clearance from the retina, its dysfunction might result in disturbance of retinal homeostasis, which triggers release of vascular endotheleal growth factor (VEGF, 2). Consecutive neovascularization of the iris (3) increases vessel permeability and penetration of gadolinium-based contrast agent into the AC (4)

We hypothesize that the correlation between AC enhancement and ON infiltration is caused by orbital glymphatic dysfunction due to reduced trans-laminar glymphatic flow caused by the tumor (Fig. 6): Glymphatic insufficiency might result in retinal accumulation of toxic metabolites and reduced nutrient supply. Disturbance of retinal homeostasis is known to cause release of vascular endothelial growth factor (VEGF) with consecutive neoangiogenesis including the iris. Neovascularization notoriously increases vessel permeability with subsequently increased penetration of GBCA into the AC following intravenous injection.

Remarkably, ON infiltration is known to be associated with increased intraocular pressure (IOP) [17], which might result from iris neovascularization with concomitant neovascular glaucoma.

### Diagnostic implication in RB

Today, if localized disease can be confirmed, RB may be treated conservatively to retain the child’s vision [3]. Therefore, initial staging aims to classify RB with respect to the predicted salvage rate of the eye with 13% of the enucleated group D eyes revealing high-risk histopathology [27, 28]. Therein, ON infiltration is a major risk factor for advanced disease with a 13% 5-year metastatic risk in patients with postlaminar ON involvement and a significant reduction in overall survival with ON infiltration depth [1, 2].

Therefore, pretreatment MRI is supposed to identify ON infiltration; however, diagnostic accuracy of MRI findings is known to be limited [5–10].

While the here-reported semiquantitative signal intensity ratio differences do not allow for calculation of a cut-off for discrimination between RB eyes with regard to ON infiltration, future studies might investigate direct imaging of the orbital GBCA distribution following intravenous injection, e.g., with heavily T2-weighted FLAIR MRI [29].

Remarkably, GBCA enhancement increased gradually with infiltration level and was greater in RB with postlaminar compared to a combined group of RB with either none or prelaminar ON infiltration (*p* = 0.06). This might be of diagnostic importance as the prognostic effect of the different ON infiltration levels remains unclear with some studies suggesting that the prognostic impact of ON infiltration might be confined to postlaminar involvement, but might not exist for prelaminar ON infiltration [1, 2].

Strengths and limitations of this study need to be acknowledged. An obvious strength of the study is the large patient cohort, the histopathology reference, and the high-resolution orbital MRI of RB as well as healthy eyes obtained with surface coils and identical sequence parameters in children under general anesthesia.

However, a final explanation for the here-introduced phenomenon cannot be concluded and the physiology of the interrelation between AC enhancement and ON infiltration remains uncertain.

Furthermore, disturbance of retinal homeostasis with consecutive neovascularization of the iris might be caused by impeded arterial perfusion / venous drainage due to affection of the central vein or artery by the tumor. However, histologic assessment revealed vascular infiltration in only two RB eyes, both of them with low ∆SIR and only one with (prelaminar) optic infiltration.

In conclusion, this study revealed a surprising link between GBCA enhancement in the anterior eye chamber and optic nerve infiltration by the retinoblastoma, which might be explained by orbital glymphatic dysfunction and serves as an example of how cerebral GBCA excretion adds diagnostic information on neurological pathologies.

## Supplementary information

Supplementary fig. e-1 Example of applied image analysis. Comparison of native (1^st^ column) and gadolinium-based contrast agent (GBCA) enhanced (2^nd^ column) T1-weighted orbital MRI in a 3-month-old girl with retinoblastoma of the left eye. The 1^st^ row presents region of interest (ROI) positioning for signal intensity ratio (SIR) calculation, the 2^nd^ row depicts the same slice without ROI placement and the 3^rd^ row gives an enlarged view of the anterior chamber (AC) that allows visual assessment of GBCA-enhancement in the AC anterior to the iris with a maximum in the iridocorneal angle (F) compared to the native scan (E). (PNG 4127 kb)

Supplementary fig. e-2 Example of applied scoring system to classify tumor size. Tumor size was classified with a 6-point ordinal scale (A-F), ranging from (A) barely identifiable retinoblastimas (RBs), affecting not more than one sixth of the globe, up to RBs preoccupying the bulbus subtotally (E) and totally (F). (PNG 6192 kb)

Supplementary fig. e-3 Comparison of anterior chamber-to-lens signal intensity ratio differences (∆SIRs) in infantile eyes affected by retinoblastoma (RB) with and without active prescan normalization filter (PDF 34 kb)

## Abbreviations

∆SIR: Difference in AC-to-lens SIR
AC: Anterior chamber
CSF: Cerebrospinal fluid
FoV: Field of view
GBCA: Gadolinium-based contrast agent
IOP: Intraocular pressure
IQR: Interquartile range
OD: Odds ratio
ON: Optic nerve
p.i.: Post injection
RB: Retinoblastoma
ROI: Region of interest
SAS: Subarachnoid space
SD: Standard deviation
SI: Signal intensity
SIR: Signal intensity ratio
TLCPD: Trans-laminar cribrosa pressure difference

## Declarations

### Guarantor

The scientific guarantor of this publication is Katerina Deike-Hofmann.

### Statistics and biometry

No complex statistical methods were necessary for this paper.

### Study subjects or cohorts overlap

Some study subjects or cohorts have been previously reported in Deike-Hofmann et al, The anterior eye chamber: entry of the natural excretion pathway of gadolinium contrast agents? Eur Radiol, 2020.

### Methodology

• retrospective

• diagnostic or prognostic study

• performed at one institution

## References

[1] Shields C, Shields JA, Baez K, Cater IR, De P (1993). Optic nerve invasion of retinoblastoma metastatic potential and clinical risk factors. Cancer..

[2] Messmer EP, Heinrich T, Höpping W, de Sutter E, Havers W, Sauerwein W (1991). Risk factors for metastases in patents with retinoblastoma. Ophthalmology..

[3] MacCarthy A, Birch JM, Draper GJ (2009). Retinoblastoma: treatment and survival in Great Britain 1963 to 2002. Br J Ophthalmol..

[4] Chintagumpala M, Chevez-Barrios P, Paysse EA, Plon SE, Hurwitz R (2007). Retinoblastoma: review of current management. Oncologist..

[5] De Graaf P, Barkhof F, Moll AC (2005). Retinoblastoma: MR imaging parameters in detection of tumor extent. Radiology..

[6] de Graaf P, Moll A, Imhof S, von der Valk M, Castelijns JA (2006). Retinoblastoma and optic nerve enhancement on MRI: not always extraocular tumour extension. Br J Ophthalmol..

[7] Schueler AO, Hosten N, Bechrakis NE (2003). High resolution magnetic resonance imaging of retinoblastoma. Br J Ophthalmol..

[8] Song KD, Eo H, Kim JH, Yoo SY, Jeon TY (2012). Can preoperative MR imaging predict optic nerve invasion of retinoblastoma?. Eur J Radiol..

[9] Li Z, Guo J, Xu X, Wang Y, Mukherji SK, Xian J (2020). Diagnosis of postlaminar optic nerve invasion in retinoblastoma with MRI features. J Magn Reson Imaging..

[10] Cui Y, Luo R, Wang R (2018). Correlation between conventional MR imaging combined with diffusion-weighted imaging and histopathologic findings in eyes primarily enucleated for advanced retinoblastoma: a retrospective study. Eur Radiol..

[11] Kim U, Rathi G, Chowdhary G, Srinavasan KG, Shanthi R, Krishna RSP (2019). Accuracy of preoperative imaging in predicting optic nerve invasion in retinoblastoma: a retrospective study. Indian J Ophthalmol..

[12] Galluzzi P, Cerase A, Hadjistilianou T (2003). Retinoblastoma: abnormal gadolinium enhancement of anterior segment of eyes at MR imaging with clinical and histopathologic correlation. Radiology..

[13] Wang X, Lou N, Eberhardt A (2020). An ocular glymphatic clearance system removes beta-amyloid from the rodent eye. Sci Transl Med..

[14] Senthil S, Dada T, Das T (2021). Neovascular glaucoma-a review. Indian J Ophthalmol..

[15] Rodrigues GB, Abe RY, Zangalli C et al (2016) Neovascular glaucoma: a review. Int J Retin Vitr 2(1). 10.1186/s40942-016-0051-x 10.1186/s40942-016-0051-x PMC5116372 27895936

[16] Beutel J, Peters S, Lüke M (2010). Bevacizumab as adjuvant for neovascular glaucoma. Acta Ophthalmol..

[17] Chawla B, Sharma S, Sen S (2012). Correlation between clinical features, magnetic resonance imaging, and histopathologic findings in retinoblastoma: a prospective study. Ophthalmology..

[18] Naganawa S, Yamazaki M, Kawai H, Sone M, Nakashima T (2011). Contrast enhancement of the anterior eye segment and subarachnoid space: detection in the normal state by heavily T 2 -weighted 3D FLAIR. Magn Reson Med Sci..

[19] Deike-Hofmann K, Reuter J, Haase R (2019). Glymphatic pathway of gadolinium-based contrast agents through the brain: overlooked and misinterpreted. Invest Radiol..

[20] Jost G, Frenzel T, Lohrke J, Lenhard DC, Naganawa S, Pietsch H (2017). Penetration and distribution of gadolinium-based contrast agents into the cerebrospinal fluid in healthy rats: a potential pathway of entry into the brain tissue. Eur Radiol..

[21] Taoka T, Jost G, Frenzel T, Naganawa S, Pietsch H (2018). Impact of the glymphatic system on the kinetic and distribution of gadodiamide in the rat brain: observations by dynamic MRI and effect of circadian rhythm on tissue gadolinium concentrations. Invest Radiol..

[22] Deike-Hofmann K, von Lampe P, Schlemmer H (2020). The anterior chamber of the eye: an overlooked entry of the natural excretion pathway of gadolinium based contrast agents?. Eur Radiol..

[23] Mathieu E, Gupta N, Ahari A, Zhou X, Hanna J, Yücel YH (2017). Evidence for cerebrospinal fluid entry into the optic nerve via a glymphatic pathway. Investig Ophthalmol Vis Sci..

[24] Killer HE, Jaggi GP, Flammer J, Miller NR, Huber AR, Mironov A (2007). Cerebrospinal fluid dynamics between the intracranial and the subarachnoid space of the optic nerve. Is it always bidirectional?. Brain..

[25] Killer HE (2013) Production and circulation of cerebrospinal fluid with respect to the subarachnoid space of the optic nerve. J Glaucoma 22. 10.1097/IJG.0b013e318293498b 10.1097/IJG.0b013e318293498b 23733131

[26] Killer HE, Laeng H, Groscurth P (1999) Lymphatic capillaries in the meninges of the human optic nerve. J Neuroophthalmol. 19(4):222–228 10608671

[27] Shields CL, Mashayekhi A, Au AK (2006). The International Classification of Retinoblastoma predicts chemoreduction success. Ophthalmology..

[28] Fabian ID, Stacey AW, Chowdhury T (2017). High-risk histopathology features in primary and secondary enucleated International Intraocular Retinoblastoma Classification Group D Eyes. Ophthalmology..

[29] Mijnders LS, Steup FW, Lindhout M, Kleij PA van der Brink WM, van der Molen AJ (2020) Optimal sequences and sequence parameters for GBCA-enhanced MRI of the glymphatic system: a systematic literature review. Acta radiol. Published online November 5, 028418512096995. 10.1177/0284185120969950 10.1177/0284185120969950 33153270

