# Supplementary figures and images for: Anterior chamber enhancement predicts optic nerve infiltration in retinoblastoma
Source: Eur Radiol. 2022 May 7;32(11):7354–64. doi: 10.1007/s00330-022-08778-4 (PMC9668776; doi:10.1007/s00330-022-08778-4)

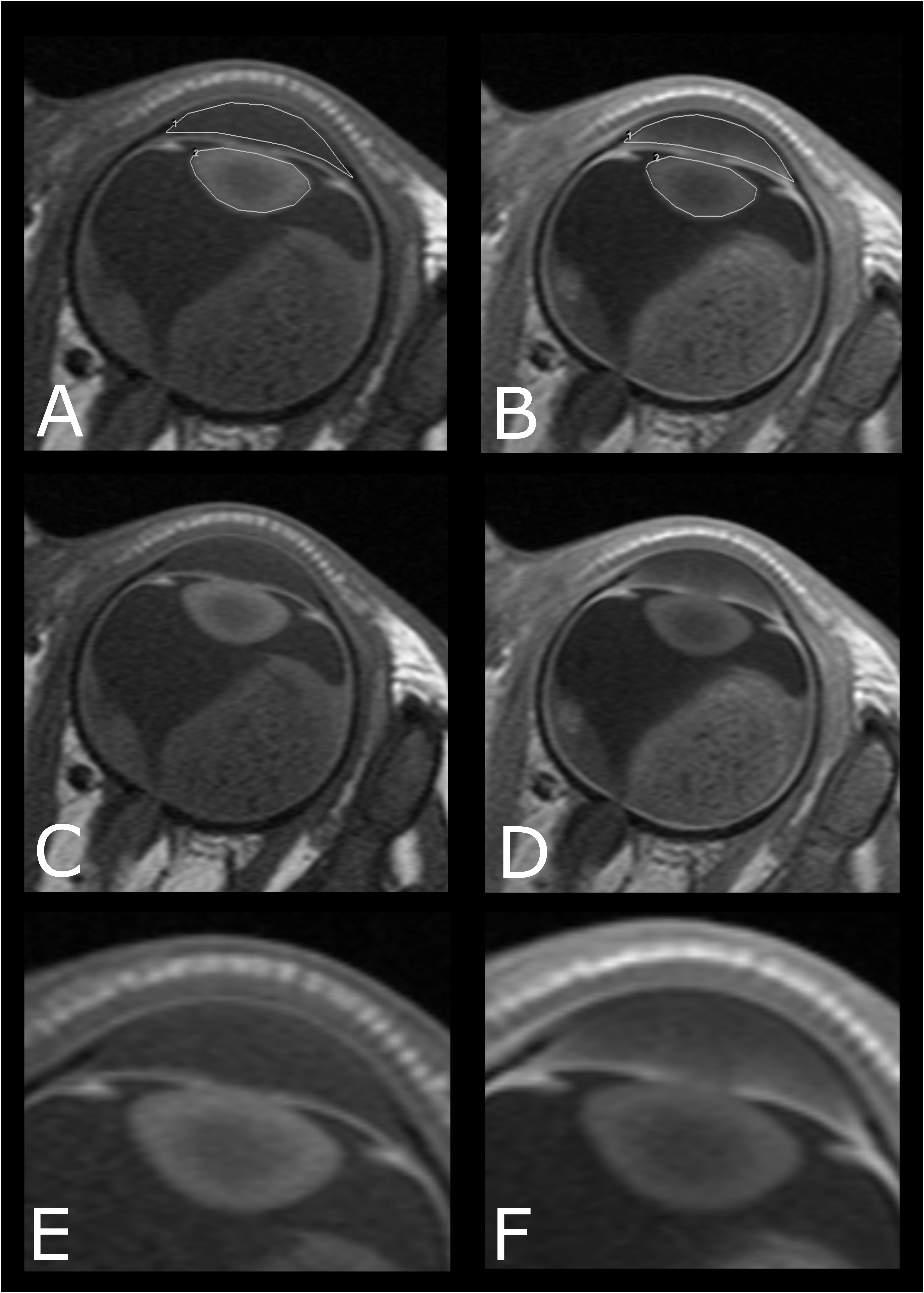

Supplement: Supplementary file 1 — Example of applied image analysis. Comparison of native (1st column) and gadolinium-based contrast agent (GBCA) enhanced (2nd column) T1-weighted orbital MRI in a 3-month-old girl with retinoblastoma of the left eye. The 1st row presents region of interest (ROI) positioning for signal intensity ratio (SIR) calculation, the 2nd row depicts the same slice without ROI placement and the 3rd row gives an enlarged view of the anterior chamber (AC) that allows visual assessment of GBCA-enhancement in the AC anterior to the iris with a maximum in the iridocorneal angle (F) compared to the native scan (E). (PNG 4127 kb) [file 330_2022_8778_MOESM1_ESM.png]

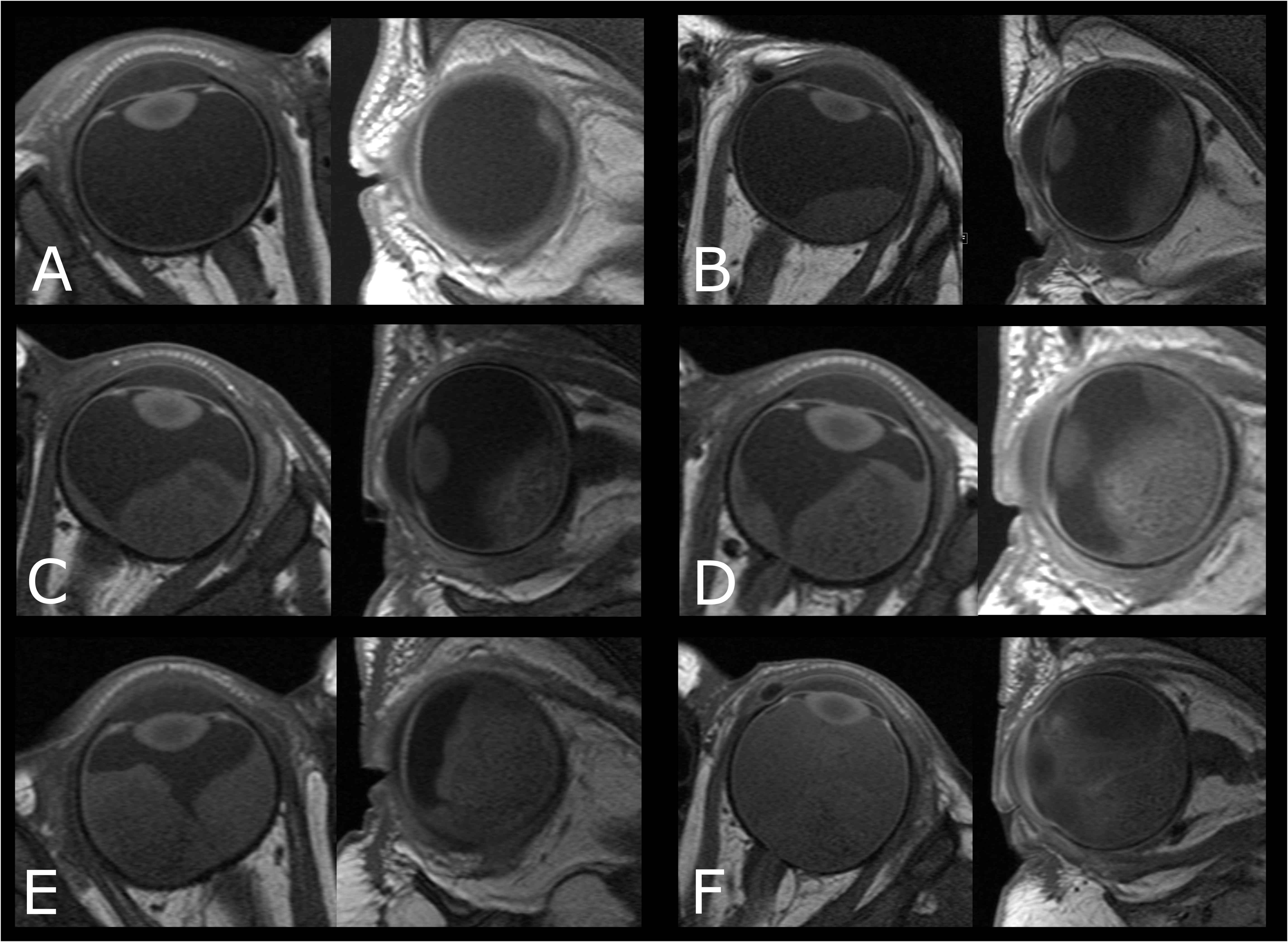

Supplement: Supplementary file 2 — Example of applied scoring system to classify tumor size. Tumor size was classified with a 6-point ordinal scale (A-F), ranging from (A) barely identifiable retinoblastimas (RBs), affecting not more than one sixth of the globe, up to RBs preoccupying the bulbus subtotally (E) and totally (F). (PNG 6192 kb) [file 330_2022_8778_MOESM2_ESM.png]
