# Supplementary material for: Anterior chamber enhancement predicts optic nerve infiltration in retinoblastoma
Source: Eur Radiol. 2022 May 7;32(11):7354–64. doi: 10.1007/s00330-022-08778-4 (PMC9668776; doi:10.1007/s00330-022-08778-4)
Supplement: Supplementary file 3 — Comparison of anterior chamber-to-lens signal intensity ratio differences (∆SIRs) in infantile eyes affected by retinoblastoma (RB) with and without active prescan normalization filter (PDF 34 kb) [file 330_2022_8778_MOESM3_ESM.pdf]

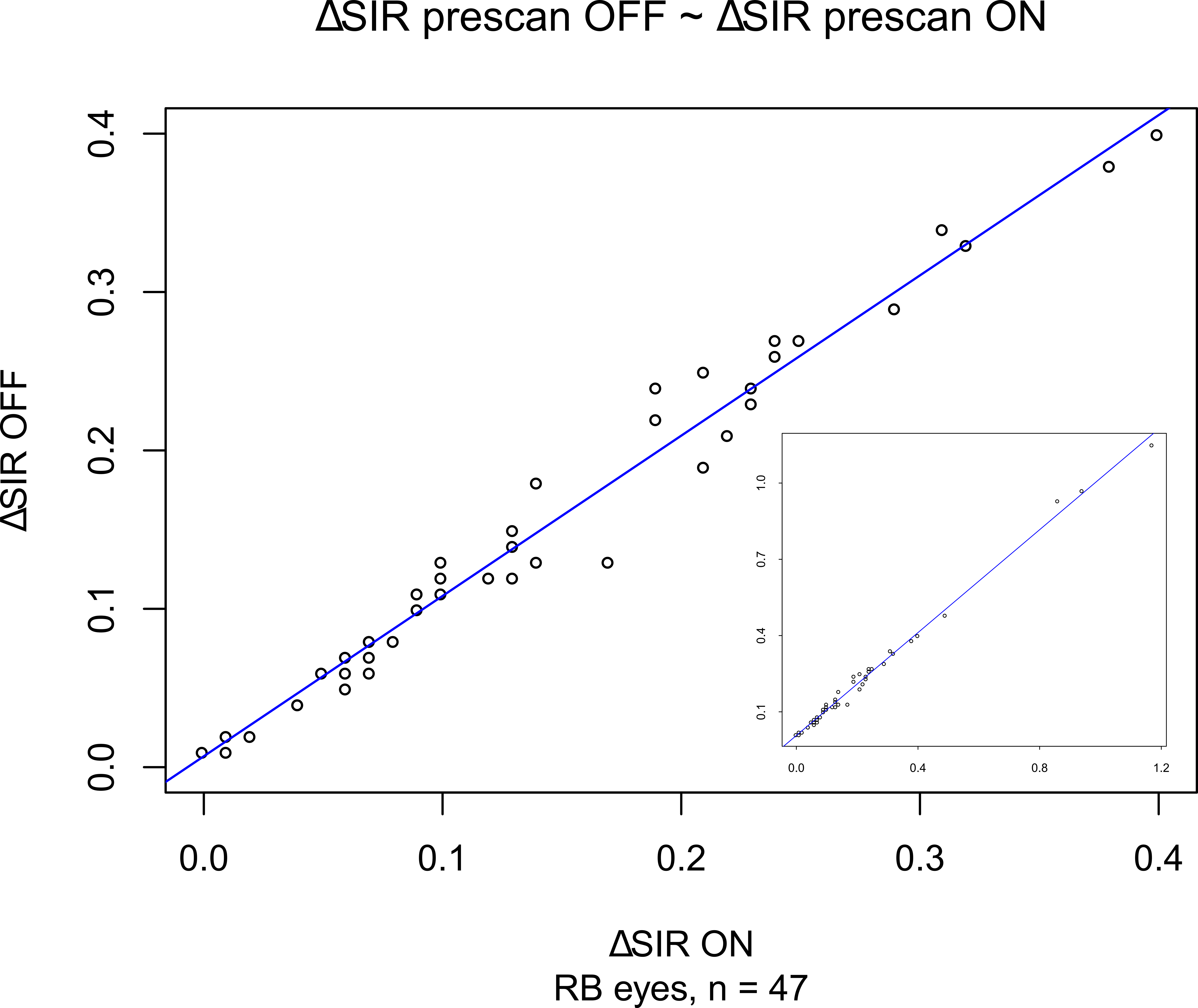

ΔSIR prescan OFF ~ ΔSIR prescan ON
ΔSIR OFF
0.0
0.1
0.2
0.3
0.4
ΔSIR ON
RB eyes, n = 47
0.1
0.4
0.7
1.0
0.0
0.4
0.8
1.2
